# Supplementary material for: Spatial, temporal and molecular dynamics of swine influenza virus-specific CD8 tissue resident memory T cells
Source: Mucosal Immunol. 2022 Feb 10;15(3):428–42. doi: 10.1038/s41385-021-00478-4 (PMC9038527; doi:10.1038/s41385-021-00478-4)
Supplement: Supplementary file 1 — Supplementary Materials [file 41385_2021_478_MOESM1_ESM.pdf]

Supplementary Materials:

Supplementary Figures

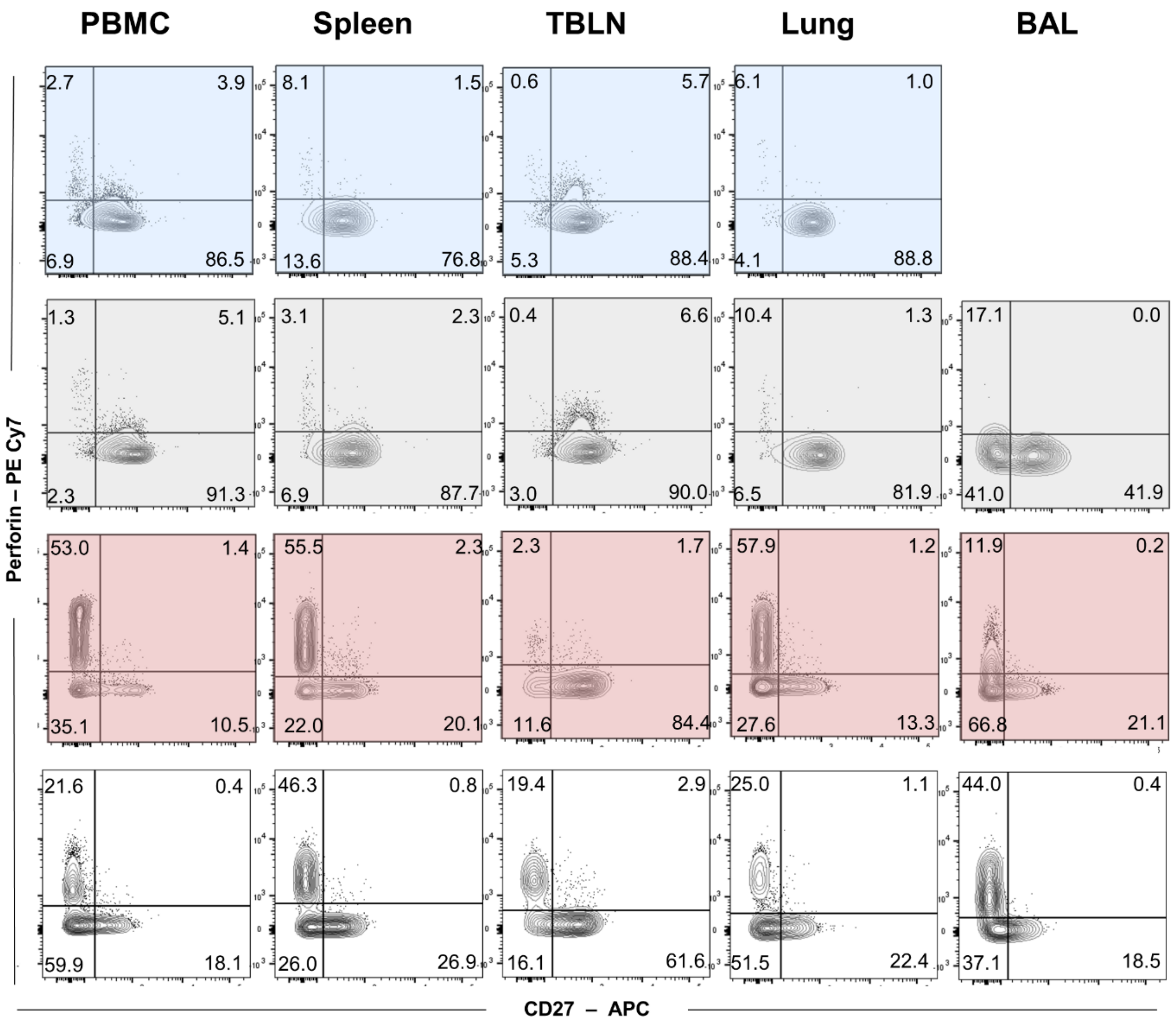

Supplementary Figure 1. Expression of perforin and CD27 in CD8 T cell subset

Representative plots showing perforin and CD27 expression in CD8 T lymphocyte subsets defined by CD45RA and CCR7 expression: naïve (CD45RA$^+$, CCR7$^+$, blue), TCM (CD45RA$^-$, CCR7$^+$, grey), TEM (CD45RA$^-$, CCR7$^-$, red panels) and TDE (CD45RA$^+$ CCR7$^-$, white panels). Each quadrant shows the mean percentage of 3 animals.

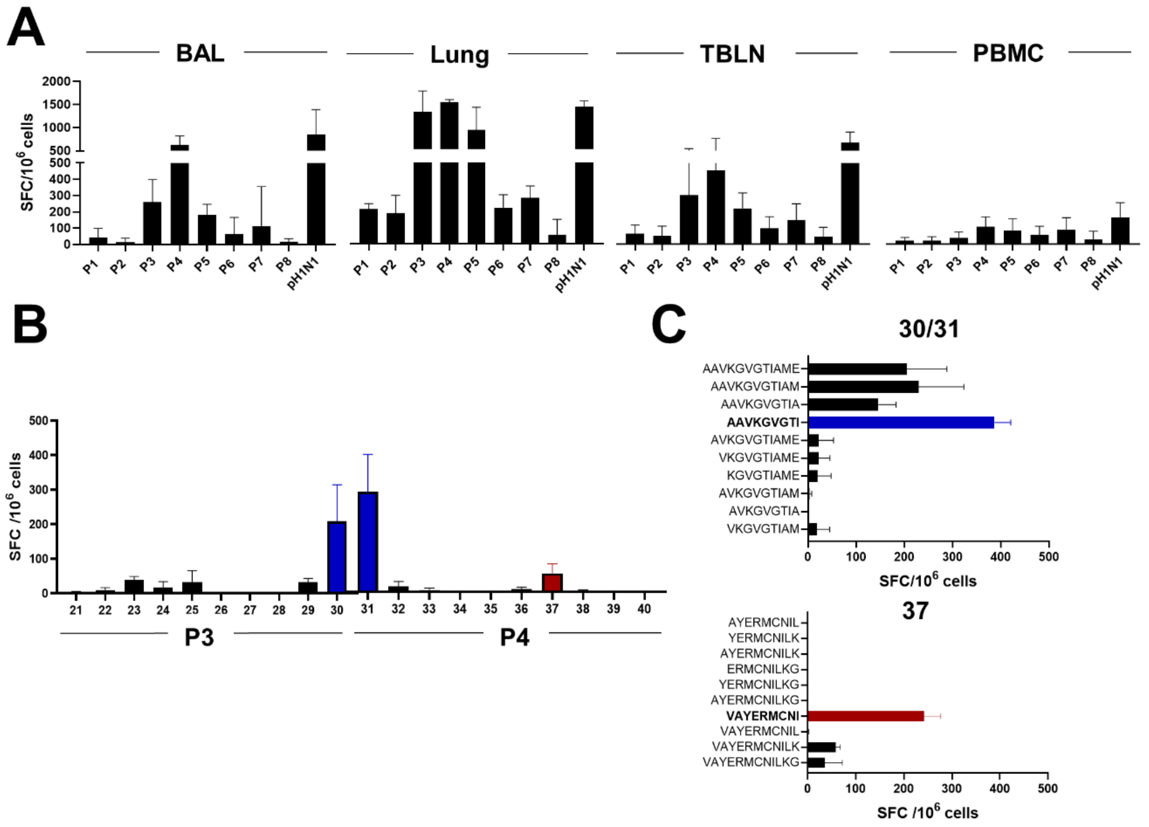

Supplementary Figure 2. Identification of NP epitopes AAV and VAY (A) Pools of 10 peptides of 18 amino acid (aa) length from the NP of H1N1pdm09 were used for an initial screen to stimulate cells isolated from BAL, lung, TBLN and PBMC at 13/14 DPI. Responses were measured by IFNg ELISpot and H1N1pdm09 (pH1N1) MOI = 1 was used as a positive control. **(B)** Spot forming cells (SFC) in TBLN after stimulation with individual peptides from pool 3 (p3) and pool 4 (p4), highlighted the peptides that subsequentially identified AAV epitope (in blue) and VAY (in red). **(C)** Minimal epitope identification (using peptides of 9 aa) from peptide 30/31 (top) and 37 (bottom) identify the AAV (in blue) and VAY (in red) epitopes. Mean and SEM of 3 animals.

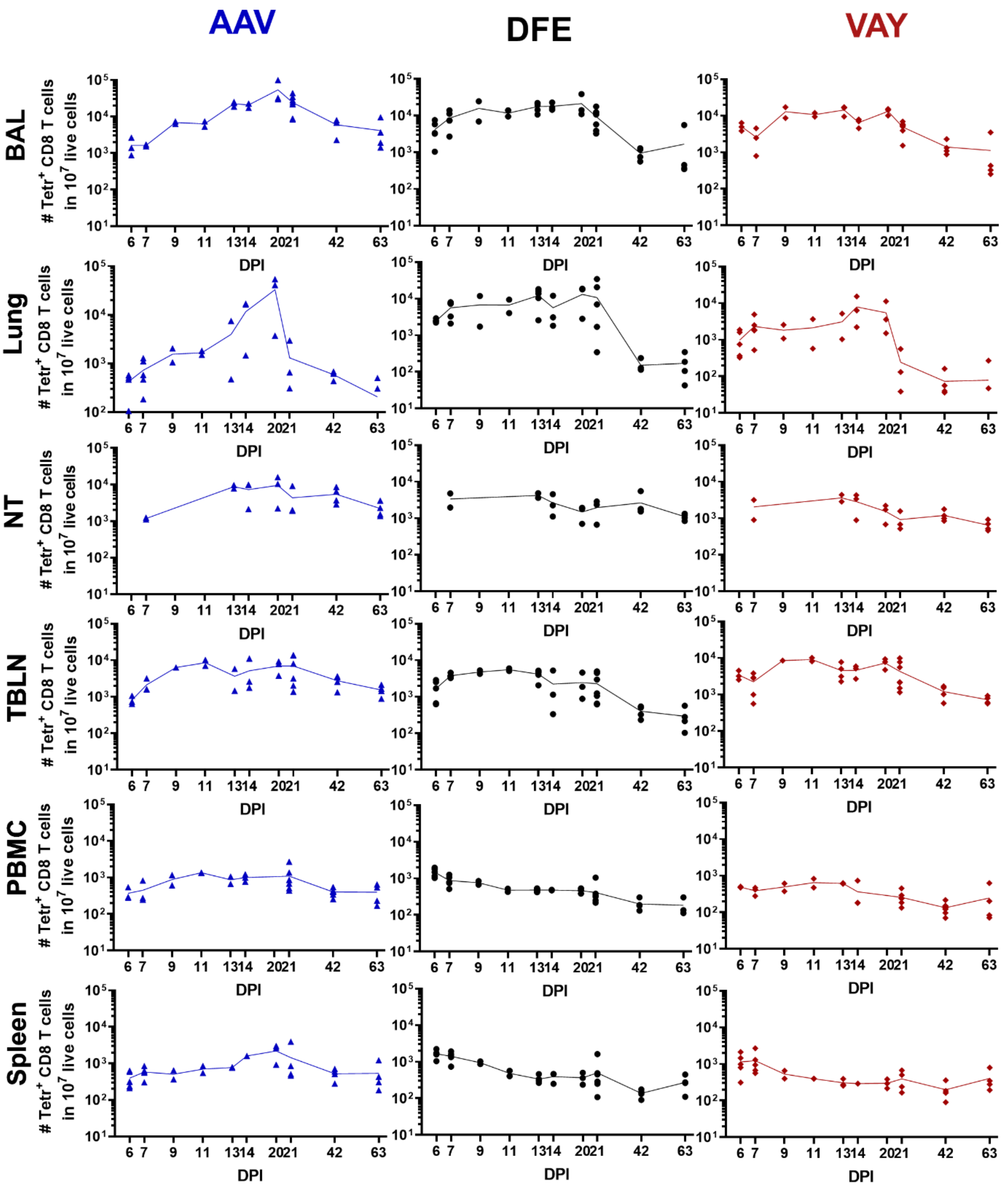

Supplementary Figure 3. Number of tetramer^{+} T cells in tissues Counts of tetramers^{+} CD8 T cells in 10 million live cells isolated from each tissues. The symbols indicates individual animals while lines connect the mean at each timepoint.

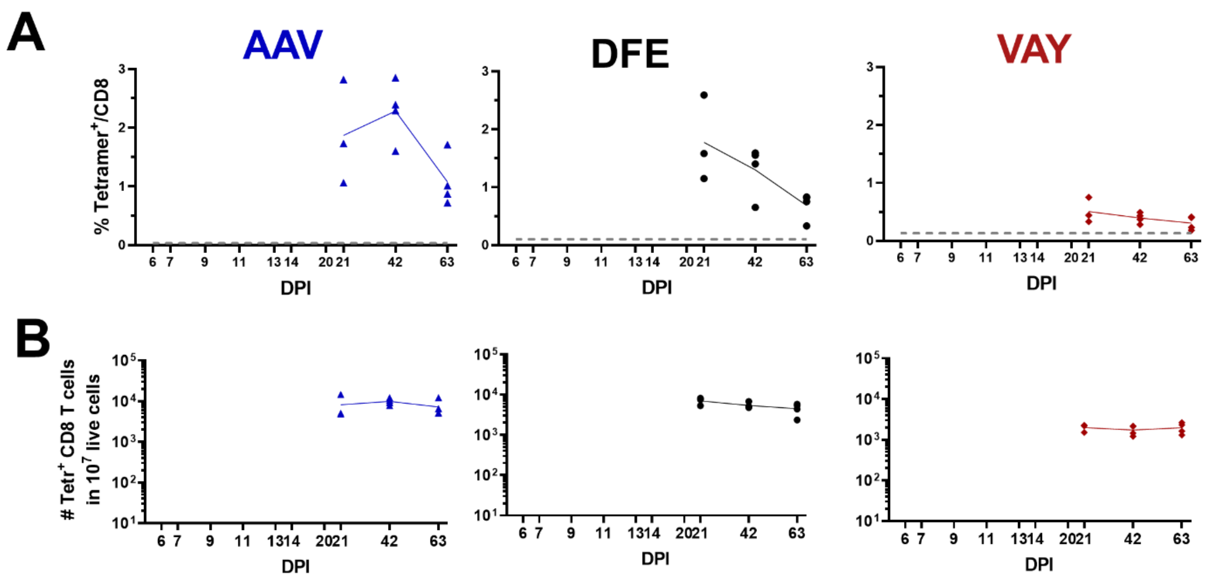

Supplementary Figure 4. **Distribution of tetramer+ cells in the trachea (A)** Percentages of DFE, VAY and AAV+ within CD8 T cell population at day 21, 42 and 63 post infection (n=4 in each time point) **(B)** Number of tetramer+ T cells in 10 million live cells. Each symbol represent an individual and the dotted line the average % of tetramer+ T cells in naïve animals.

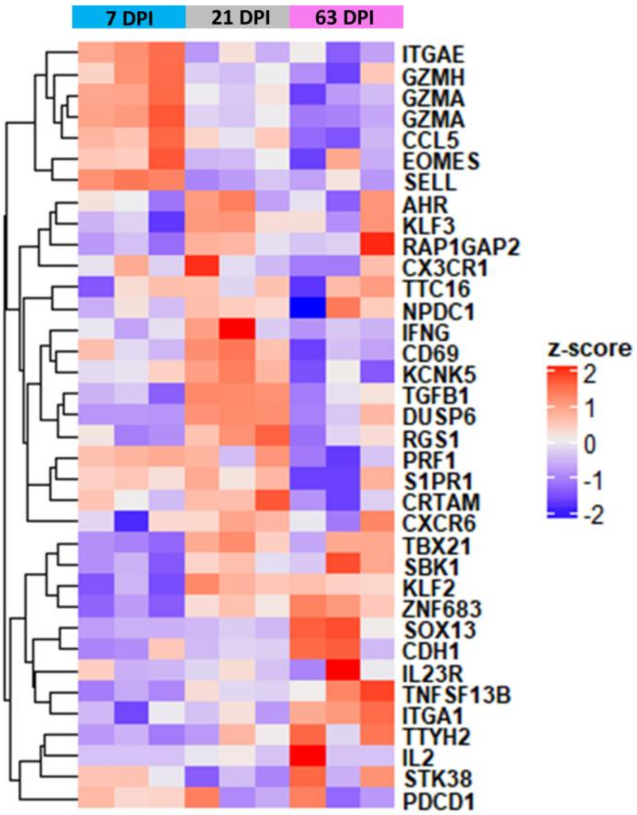

Supplementary Figure 5. Gene expression of tissue resident memory T cells features. Heatmap of selected genes related to tissue resident memory T cells in BAL at 7, 21 and 63 days post infection (n=3).

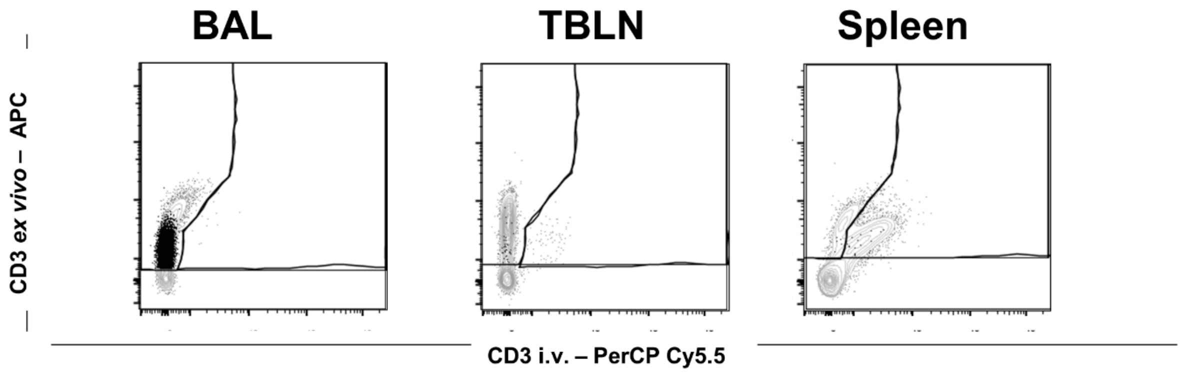

Supplementary Figure 6. CD3 infusion for the identification of tissue resident memory T cells. Representative FACS plots showing CD3 staining of DFE^{+} T cells (in back) and total live lymphocytes (in grey) in BAL, TBLN and spleen of S-FLU aer immunized animal 3 weeks post boost.

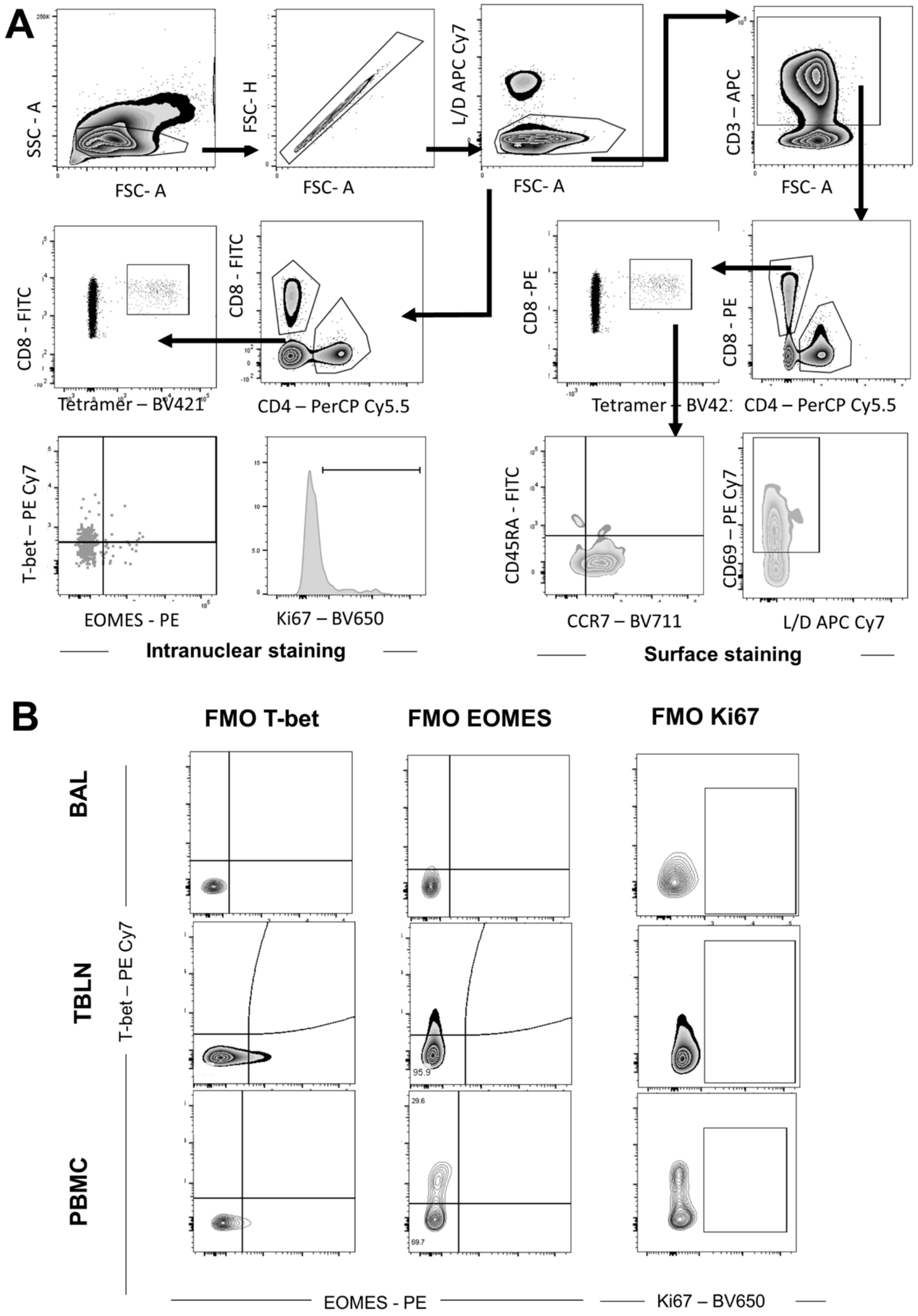
A
SSC - A
FSC- A
FSC- H
FSC- A
L/D APC Cy7
FSC- A
CD3 – APC
FSC- A
CD8 - FITC
Tetramer – BV421
CD8 - FITC
CD4 – PerCP Cy5.5
CD8 -PE
Tetramer – BV421
CD8 - PE
CD4 – PerCP Cy5.5
T-bet – PE Cy7
EOMES - PE
Ki67 – BV650
CD45RA - FITC
CCR7 – BV711
CD69 – PE Cy7
L/D APC Cy7
Intranuclear staining
Surface staining
B
FMO T-bet
FMO EOMES
FMO Ki67
BAL
TBLN
PBMC
T-bet – PE Cy7
95.9
29.6
69.7
EOMES - PE
Ki67 – BV650

Supplementary Figure 7. Gating strategy and controls. (A) Representative FACS plots (from PBMC) showing the gating strategy for intranuclear staining (panels on the left) and surface marker staining (on the right.) **(B)** Fluorescence minus one (FMO) controls for transcriptional factors in the different tissues

Supplementary Tables

Supplementary Table 1. Comparison of models describing the change over time in the proportion of $CD8^+$ T cells specific for each tetramer in different tissues. (The best fit model is shown in bold.)

	model*			RSS†	df†	P†
	y_0	y_{max}	t_{max}			
1	tissue, tetramer	tissue, tetramer	tissue, tetramer	0.0446	989	-
2	tetramer	tissue, tetramer	tissue, tetramer	0.0475	1004	<0.001
3	tissue, tetramer	tetramer	tissue, tetramer	0.0724	1004	<0.001
4	tissue, tetramer	tissue, tetramer	tetramer	0.0485	1001	<0.001
**5**	**tissue**	**tissue, tetramer**	**tissue, tetramer**	**0.0454**	**1001**	**0.12**
6	tissue, tetramer	tissue	tissue, tetramer	0.0558	1001	<0.001
7	tissue, tetramer	tissue, tetramer	tissue	0.0512	1001	<0.001
8	neither	tissue, tetramer	tissue, tetramer	0.0477	1006	<0.001

* dependence of model parameters on tissue or tetramer

† RSS - residual sum-of-squares; df - degrees of freedom; P - *P*-value for *F*-tests comparing the residual deviance for models 2-7 with that for model 1 or the residual deviance for model 8 with that for model 5

Supplementary Table 2. Comparison of models describing the change over time in the relative proportion of $CD8^+$ T cells specific for each tetramer in different tissues. (The best fitting model is shown in bold.)

	model*				RSS†	df†	P†
	K_1	K_2	*b*	*d*			
1	varies	varies	varies	varies	4.62	116	-
2	common	varies	varies	varies	13.40	126	<0.001
3	varies	common	varies	varies	5.18	126	0.19
4	varies	varies	common	varies	53.13	126	<0.001
5	varies	varies	varies	common	21.24	126	<0.001
**6**	**common**	**common**	**varies**	**varies**	**5.38**	**136**	**0.90**
7	varies	common	common	varies	5.54	136	0.57
8	varies	common	varies	common	5.48	136	0.69
9	common	common	common	varies	6.44	146	0.005
10	common	common	varies	common	8.34	146	<0.001

* common - parameter independent of tissue; varies - parameter varies amongst tissues
† RSS - residual sum-of-squares; df - degrees of freedom; P - *P*-value for *F*-tests comparing the residual deviance for (i) models 2-5 with that for model 1; (ii) models 6-8 with that for model 3; or (iii) models 9-10 with that for model 6

Supplementary Table 3. Top 15 most significant KEGG pathways upregulated in 21DPI vs 7DPI comparison.

Gene Set	Pathway	Normalised enrichment score	FDR
ssc04640	Hematopoietic cell lineage	1.782321698	0
ssc04658	Th1 and Th2 cell differentiation	1.733043263	0
ssc04659	Th17 cell differentiation	1.695568699	5.99E-04
ssc05164	Influenza A	1.700025793	7.99E-04
ssc04668	TNF signaling pathway	1.68935987	9.59E-04
ssc05410	Hypertrophic cardiomyopathy (HCM)	1.670102628	0.00137
ssc04010	MAPK signaling pathway	1.684975448	0.001398
ssc05202	Transcriptional misregulation in cancer	1.654904874	0.001648
ssc05418	Fluid shear stress and atherosclerosis	1.64832379	0.002264
ssc04060	Cytokine-cytokine receptor interaction	1.637482379	0.003487
ssc05169	Epstein-Barr virus infection	1.634538696	0.003595
ssc04145	Phagosome	1.638094341	0.003715
ssc04380	Osteoclast differentiation	1.623347811	0.004623
ssc04612	Antigen processing and presentation	1.624667513	0.004794
ssc04350	TGF-beta signaling pathway	1.607515815	0.007416

Supplementary Table 4. Comparison of models describing the change over time in the proportion of T cells specific for each tetramer expressing different cell surface markers in different tissues. (The best fitting models are shown in bold.)

	model*		RSS†				df†	P†			
	y_0	*d*	Ki67	T-bet	EOMES	CD69		Ki67	T-bet	EOMES	CD69
1	tissue, tetramer	tissue, tetramer	**1.792**	**1.864**	0.884	**2.434**	262	-	-	-	-
2	tetramer	tissue, tetramer	2.027	2.258	1.193	2.870	268	<0.001	<0.001	<0.001	<0.001
3	tissue, tetramer	tetramer	2.027	2.369	2.658	2.999	268	<0.001	<0.001	<0.001	<0.001
4	tissue	tissue, tetramer	2.722	2.276	0.973	2.776	268	<0.001	<0.001	<0.001	<0.001
5	tissue, tetramer	tissue	2.533	2.408	**0.889**	3.228	268	<0.001	<0.001	0.96	<0.001
6	tissue, tetramer	common	not fitted	not fitted	1.061	not fitted	270	-	-	<0.001	-
7	tissue, tetramer	PBMC: zero; TBLN, BAL: tetramer	not fitted	not fitted	not fitted	2.506	265	-	-	-	0.05

* dependence of model parameters on tissue or tetramer

† RSS - residual sum-of-squares; df - degrees of freedom; P - *P*-value for *F*-tests comparing the residual deviance for models 2-5 with that for model 1 or the residual deviance for model 6 with that for model 5

Supplementary Table 5. List of antibodies used.

Antigen	Clone	Isotype	Fluorochrome	Source of primary Ab	Details of secondary Ab
CD4	74-12-4	IgG2b	PerCP-Cy5.5	BD Biosciences	
CD8β	PPT23	IgG1	FITC	Bio-Rad Laboratories	
CD8β	PPT23	IgG1	PE	Bio-Rad Laboratories	
TNF	MAb11	IgG1	BV421	BioLegend	
IFNγ	P2G10	IgG1	APC	BD Biosciences	
IL-2	A150D 3F1 2H2	IgG2a	PE-Cy7	ThermoFisher	rat-anti-mouse, IgG2a, BioLegend
CCR7	3D12	IgG2a	BV711	BD Biosciences	
CD45RA	MIL13	IgG1	FITC	Bio-Rad Laboratories	
Annexin V	N/A	N/A	BV510	BioLegend	
CD69	01-14-22-51	IgG2b	PE Cy7	Kyoto Institute of Nutrition & Pathology (Hayashi et al. 2018)	Goat-anti-mouse, BioLegend
EOMES	WD1928	IgG1	PE	eBioscience	
T-bet	eBio4B10	IgG1	PE Cy7	eBioscience	
Ki67	B56	IgG1	BV650	BD Biosciences	
Perforin	δG9	IgG2b	Purified		Lightning-Linked PE-Cy7, antibody labelling kit, Novus Bio
CD27	B30C7	IgG1	APC	Bio-Rad Laboratories	
CD14	REA599	IgG1	PE	Miltenyi Biotec	
CD172a	BL1H7	IgG1	PE	Bio-Rad Laboratories	
Live/Dead Marker	N/A	N/A	Fixable Near-IR	Life Technologies	
